# Rift Valley Fever during Rainy Seasons, Madagascar, 2008 and 2009

**DOI:** 10.3201/eid1606.091266

**Published:** 2010-06

**Authors:** Soa Fy Andriamandimby, Armand Eugène Randrianarivo-Solofoniaina, Elisabeth M. Jeanmaire, Lisette Ravololomanana, Lanto Tiana Razafimanantsoa, Tsanta Rakotojoelinandrasana, Josette Razainirina, Jonathan Hoffmann, Jean-Pierre Ravalohery, Jean-Théophile Rafisandratantsoa, Pierre E. Rollin, Jean-Marc Reynes

**Affiliations:** Institut Pasteur, Antananarivo, Madagascar (S.F. Andriamandimby, T. Rakotojoelinandrasana, J. Razainirina, J.Hoffmann, J.-P. Ravalohery, J.-T. Rafisandratantsoa, J.-M. Reynes); Ministère de la Santé et du Planning Familial, Antananarivo (A.E. Randrianarivo-Solofoniaina, L. Ravololomanana); Food and Agriculture Organization, Antananarivo (E.M. Jeanmaire); Ministère de l’Agriculture, d’Elevage et de la Pêche, Antananarivo, (L.T. Razafimanantsoa); Centers for Disease Control and Prevention, Atlanta, Georgia, USA (P.E. Rollin); 1 Current affiliation: Centre Pasteur du Cameroun, Yaounde, Cameroon.

**Keywords:** Rift Valley fever virus, arbovirus, outbreak, Bunyaviridae, Madagascar, viruses, research

## Abstract

During 2 successive rainy seasons, January 2008 through May 2008 and November 2008 through March 2009, Rift Valley fever virus (RVFV) caused outbreaks in Madagascar. Human and animal infections were confirmed on the northern and southern coasts and in the central highlands. Analysis of partial sequences from RVFV strains showed that all were similar to the strains circulating in Kenya during 2006–2007. A national cross-sectional serologic survey among slaughterhouse workers at high risk showed that RVFV circulation during the 2008 outbreaks included all of the Malagasy regions and that the virus has circulated in at least 92 of Madagascar’s 111 districts. To better predict and respond to RVF outbreaks in Madagascar, further epidemiologic studies are needed, such as RVFV complete genome analysis, ruminant movement mapping, and surveillance implementation.

## Abstract 2

The virus reemerged during an outbreak in Madagascar in 2008.

Rift Valley fever virus (RVFV) belongs to the family *Bunyaviridae*, genus *Phlebovirus*, and was first isolated in 1930 during an investigation of a large epizootic in Kenya. Virions are enveloped and contain 3 single-stranded RNA genome segments designated large (L), medium (M), and small (S) coding for the viral proteins.

Rift Valley fever (RVF) is an arthropod-borne zoonosis; it affects ruminants and is characterized by high rates of abortion and death in young and adult animals. Economic consequences of this disease can be devastating. In humans, symptoms are usually mild, but in severe cases hemorrhage, meningoencephalitis, retinopathy, and death can occur. RVFV has been detected across Africa, from Senegal to Madagascar and from Egypt to South Africa. In 2000, RVFV reached the Arabian Peninsula (*1*).

Animals are typically infected before humans. RVFV is transmitted between ruminants primarily by bites of mosquitoes of numerous genera and species. Humans can also be infected by these vectors as well as by contact or inhalation of aerosols generated when handling sick or dead infected animals or their fresh tissues. Treatment of human patients is based on signs and symptoms; a commercial vaccine is available for animals only. RVFV outbreaks are periodic and occur every 10–15 years. Between epidemics, the virus is believed to be maintained through vertical transmission by mosquitoes of the genus *Aedes*. Outbreaks are closely linked to climate variations, especially widespread increased rainfall, that favor the hatching of mosquito eggs and the subsequent emergence of a large number of adult mosquitoes (*2*). Moderate or large outbreaks that have been documented in the Horn of Africa (1989, 1997–1998, 2006–2007) were associated with widespread rainfall. For the purpose of predicting RVF outbreaks in this area, a model based on several satellite-derived observations has been proposed (*3*).

RVFV has also been detected in Madagascar. The first isolate was obtained from mosquitoes caught during the March 1979 rainy season in a forest area in the Moramanga district (no. 514; Appendix Figure), 120 km east of Antananarivo (*4*). Then in March 1990, an RVF epizootic occurred in Fenoarivo Atsinana (district 509) on the east coast, where an abnormally high incidence of abortions and disease in humans was reported (*5*,*6*). A year later, from February through April 1991, RVFV was responsible for abortions and deaths of cattle in the central highlands. Human cases were also confirmed (*7*,*8*). After the outbreaks of RVF in 2006 and 2007 in the Horn of Africa (*9*), 17 years later, the virus was again detected in Madagascar during a major outbreak. We report some features of this outbreak and the results of preliminary molecular characterization of the circulating virus. We also performed a nationwide serosurvey to determine the range of past and recent RVFV circulation.

## Materials and Methods

### Human Surveillance Systems

In 1996, in accordance with World Health Organization resolution AFR/RC43/R7, the Integrated Diseases Surveillance and Response system was implemented by the Direction des Urgences et de la Lutte contre les Maladies (DULM) from the Malagasy Ministry of Health. Hemorrhagic fevers are among the reportable diseases. Each week, basic health centers and hospitals (district and regional) must notify DULM about cases or absence of cases.

In addition, in 2007 the DULM set up, in collaboration with the Institut Pasteur de Madagascar (IPM), a sentinel surveillance system including 19 clinical sites (basic health centers). Each site reports daily to the central level (DULM and IPM) the number of patients, persons with fever, confirmed malaria cases, suspected arboviral disease cases, and suspected influenza cases. Suspected arboviral disease cases were defined as cases in patients with axillary temperature >37.5°C and >2 of the following signs: headache, myalgia, arthralgia, retro-orbital pain, and cutaneous rash or hemorrhagic signs. Of the 19 centers, 4 are also biological surveillance sites. Serum samples from patients with suspected arboviral disease are sent weekly in liquid nitrogen to the IPM.

During the outbreak, a suspected case of RVF was defined as illness in a person with a hemorrhagic syndrome and history of fever or as encephalitis and a dengue-like syndrome after exposure to sick or dead ruminants or exposure to ruminants in a village where sick or dead animals had been reported since detection of the first cases.

### Laboratory Diagnosis

#### Serologic Assays

RVFV immunoglobulin (Ig) M and IgG ELISAs were performed as described previously (*10*). After heat and detergent inactivation, serum samples were tested by anti-RVFV–specific IgM and IgG ELISAs. The assays were completed by using inactivated RVFV-infected Vero E6 cell antigens and uninfected Vero E6 cell antigens; 4 dilutions of each serum (1:100, 1:400, 1:1,600, 1:6,400) were used. Titers and the cumulative sum of optical densities of each dilution (SUM_OD_) minus the background absorbance of uninfected control Vero E6 cells (adjusted SUM_OD_) were recorded. Results of the assays for serum samples were considered positive only if the adjusted SUM_OD_ and titer were above preestablished conservative cutoff values, which were set for IgM ELISA (>0.75 and >400, respectively) and IgG ELISA (>0.95 and >400, respectively). A probable RVFV infection was one in which no RVFV was detected but antibodies were detected. Infection was considered recent when IgM against RVFV was detected and past when only IgG against RVFV (no IgM against RVFV) was detected.

#### Virologic Assays

Virus isolation was performed on mosquito cell lines (AP61 and Vero E6) by using acute-phase serum samples (diluted 1:10), and virus identification was performed by an indirect immunofluorescence assay that used pools of mouse immune ascetic fluids (*11*). These fluids reacted against several arboviruses previously isolated from Madagascar, including RVFV.

#### Molecular Assays

Liver or spleen specimens (50–100 mg) from dead animals with suspected RVF were homogenized at a dilution of 1:10 in culture medium containing 30% fetal bovine serum. The supernatant was collected after centrifugation. Viral RNA was extracted from the serum samples of patients and animals suspected of having infection by using the QIAamp Viral RNA Mini Kit (QIAGEN GmbH, Hilden, Germany) and from organ supernatants by using TRIzol LS reagent (Invitrogen, Carlsbad, CA, USA) according to the manufacturers’ instructions.

The molecular detection of the virus was performed by using a nested reverse transcription–PCR (RT-PCR) described by Sall et al. (*12*) or a real-time RT-PCR described by Weidmann et al. (*13*). The minimum level of detection was 25 transcript RNA copies per assay. A confirmed RVFV infection was an infection in which RVFV was isolated or RVFV RNA was detected.

### Molecular Characterization

Parts of the S, M, and L segments of RVFV were amplified and sequenced. S-amplified product (portion of nonstructural protein) was obtained by using the nested RT-PCR technique published by Sall et al. (*12*). M-amplified product (portion of *G2*) was obtained by using RT-PCR primers M-F675 (5′-ACCATCATTGCAAAGGCTGA-3′) and M-R1645 (5′-GCCATGTGAACCCCTATGTC-3′) and nested PCR primers MRV1a and MRV2g used by Sall et al. (*14*). L-amplified product (portion of L) was obtained by using RT-PCR primers L-F4209 (5′-GCGCATTGCAGAGAAAGTC) and L-R5113 (5′-CAACGTGATCACCATCTAGAAA-3′) and nested PCR primers L-F4273 (5′-TGTAAAGTCATGGCCTCAGC-3′) and L-R4878 (5′-CATCCGGGAGAAATTGTCA-3′). M-F675, M-R1645, L-F4209, L-R5113, L-F4273, and L-R4878 were designed to obtain M- and L-nested PCR products >600 bp; Primer3Plus software (*15*) was used according to 33 published complete RVFV M or L sequences (*16*).

Amplification products were sequenced on both strands by Cogenics (Meylan, France). Unverified sequences and chromatograms were compared and corrected when needed. Sequences from the same segment were compared when aligned, and a phylogenetic analysis was conducted by using MEGA version 4 software (*17*). The partial S, M, and L sequences obtained in this study are available from GenBank under accession nos. GQ443126–GQ443256.

### Nationwide Serologic Survey

A nationwide cross-sectional serologic survey was conducted among persons at risk for RVF. In all 111 districts of Madagascar (Appendix Figure), persons were invited to participate in the survey if they had worked in slaughterhouses within the administrative center of the district since 2007, had been exposed to fresh meat or blood of ruminants, and had been residents of the district when they started this work. The study was approved by the Malagasy National Ethical Committee. From those who gave written informed consent, 5 mL of blood was collected into red-top tubes. Samples; informed consent forms; and data sheets recording age, sex, location, date of sampling, and criteria of sampling were sent to the IPM, where RVFV IgG and IgM ELISAs were performed.

## Results

### Outbreaks

**Figure 1:**
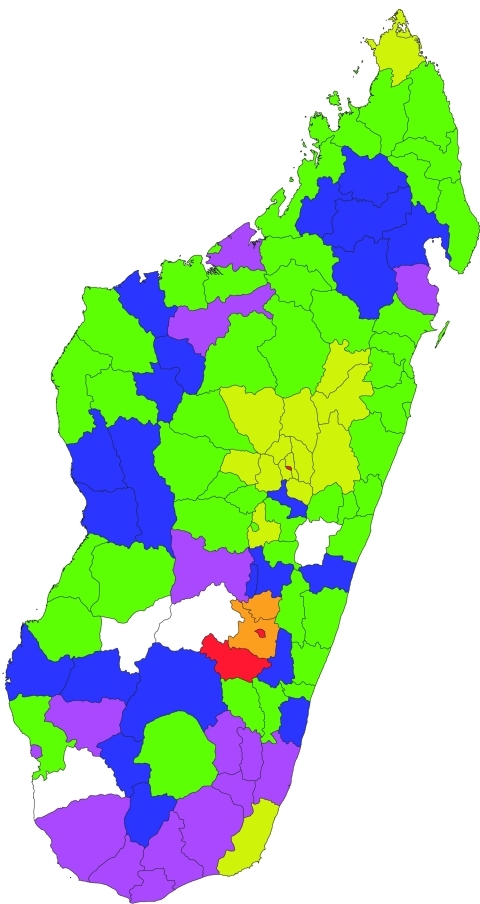
Distribution of Rift Valley fever in the 111 administrative districts in Madagascar, 2008 and 2009.  Districts with laboratory-diagnosed confirmed or probable cases in humans and/or animals are indicated by yellow (2008), orange (2009), or red (both years). In districts without confirmed or probable cases, antibody data for Rift Valley fever virus immunoglobulin (Ig) levels in serum samples from at-risk professionals are indicated by green (IgM positive only), blue (IgG positive, IgM negative), or violet (IgG and IgM negative). No samples were received from districts shown in white.

The first RVF case of 2008 was detected during routine activity of the biological surveillance sentinel center in Tolagnaro city (district no. 614) in southern Madagascar (Figure 1; Appendix Figure). The virus was isolated on AP61 and Vero E6 cells from an acute-phase serum sample collected on January 30, 2008, from a pregnant woman who had had a dengue-like syndrome for 2 days. Retrospective investigation showed that each day she had collected fresh meat from a local slaughterhouse to make and sell meat kebabs. On February 5, 2008, the DULM received an alert through the Integrated Diseases Surveillance and Response system. Cases of hemorrhagic fever and dengue-like fever, associated with deaths of farmers and with abnormal mortality rates for zebus, were reported from the Anjozorobe district (no. 107), 80 km north of Antananarivo. IgM against RVFV was detected in 16 of 23 persons sampled on February 9, 2008. These patients had been ill 1–3 weeks earlier. Retrospective investigation suggested that the virus had been circulating among livestock since December 2007.

Consequently, the DULM encouraged notification and sampling of RVF suspected cases by the basic health centers and district and regional hospitals. Overall, from January 28 through June 15, 2008, when the active surveillance was stopped, 476 suspected cases (19 of which were fatal) from 15 districts, mostly from the central highlands, were reported to the DULM.

Serum samples were received at IPM from 134 persons with suspected cases who lived in 16 districts: 36 cases were confirmed, and 31 were considered probable. Laboratory analysis results were negative for the other 67. Most of the persons with probable and confirmed cases had occupational risk; 54 were farmers and 5 were butchers. Additionally, serum, organs, or fetuses were received from 119 animals sampled by the Direction des Services Vétérinaires in 19 districts. Of the 119, cases were confirmed for 15, considered probable for 7, and were negative for the other 97. Overall, RVFV confirmed and probable cases were identified in 19 districts (Figure 1). The last laboratory-confirmed case consisted of a fatal hemorrhagic disease that began on May 23, 2008, in a person from the Amparafaravola district (no. 504), near Lake Aloatra, 180 km north of Antananarivo.

Six months later, during the rainy season (October 2008–May 2009), an alert was launched by the Direction des Services Vétérinaires when abnormally high death rates among cattle were reported from Fianarantosoa-I (no. 301) and Fianarantsoa-II (no. 302) districts (located in the central highlands). Livers from 3 cows sampled on November 28, 2008, and sent to IPM were infected with RVFV. Suspected human RVF cases from this area were confirmed. Overall, from December 1, 2008, through May 15, 2009, 236 suspected cases (including 7 deaths) in humans, were reported to the DULM: 1 from Antananarivo (no. 101) and the rest from 4 neighboring districts: 74 cases from Fianarantsoa-I (no. 301), 152 from Fianarantsoa-II (no. 302), 4 from Ambohimahasoa (no. 305), and 5 from Ambalavao (no. 303). Of the 47 suspected cases sampled, 10 were confirmed and 9 were probable. Laboratory analysis results were negative for the other 28 cases. More than half of the 19 persons with confirmed and probable cases were at high risk: 8 were farmers, 1 a butcher, 1 a veterinarian, and 1 (from Antananarivo) a cattle trader who transported animals from the south through the infected area to Antananarivo. Additionally, confirmed or probable infection was detected in serum samples or livers from 24 ruminants among 88 sampled in Fianarantsoa-I and Fianarantsoa-II districts during the period. The last laboratory-confirmed RVFV case was in a person who had hemorrhagic manifestations and lived in the Ambalavao district (no. 303); disease onset was March 13, 2009.

### Genetic Analysis

Verified partial S sequences encompassing 627 nt (positions 49–675 of the coding domain) were obtained from 46 of the 51 RVFV-positive samples collected during the January–May 2008 outbreak. These 46 samples originated from 14 of the 17 districts where the virus was detected, including the 3 main areas concerned (northern coast, southern coast, and central highlands) (Figure 1; Table). Of these 46 samples, 34 were collected from humans, 10 from cattle, 1 from a sheep, and 1 from a goat (Table). The maximum nucleotide percent identity difference between the 46 sequences was low (0.96%; 6/627). Of 11 haplotypes of sequences detected, 1 included 35 identical sequences from strains originating from 5 districts (central highlands). At the protein level, the maximum percent identity difference was 1.4% (3/209): 7 haplotypes of sequences were detected (data not shown). The virus sequences of cattle strains were identical to those of human strains in the 2 districts where RVFV strains had been obtained from cattle and humans. Phylogenetic analysis, using the neighbor-joining method with the Kimura 2-parameter model, was undertaken for the 11 Madagascar sequences representative of all the diversity, the corresponding part of the 33 complete S sequences described by Bird et al. (*16*), and the 6 sequences representative of the lineages Kenya-1, -1a and -2 recently described during the 2006–2007 Kenya outbreak (*18*). Although the bootstrap values did not support an unambiguous phylogenetic classification, the analysis showed that the Madagascar sequences were close to the Kenya sequences obtained from strains circulating in 2006–2007, especially to Kenya-1 and -1a lineages.

**Table:** Features of 46 sequenced Rift Valley fever viruses, Madagascar, 2008–2009

Code	Species	Sample type	Sampling date	District of sampling	Coding sequences		
					Small	Medium	Large
212-08	Human	Serum	2008 Jan 30	Tolagnaro	Yes	Yes	Yes
406-08	Bovine	Fetal liver	2008 Feb 20	Miarinarivo	Yes	Yes	Yes
413-08	Bovine	Fetal liver	2008 Feb 20	Antsirabe II	Yes	Yes	Yes
427-08	Human	Serum	2008 Feb 22	Anjozorobe	Yes	Yes	Yes
428-08	Human	Serum	2008 Feb 15	Anjozorobe	Yes	Yes	Yes
435-08	Bovine	Serum	2008 Feb 22	Anjozorobe	Yes	No	Yes
437-08	Bovine	Serum	2008 Feb 22	Anjozorobe	Yes	No	No
448-08	Human	Serum	2008 Feb 26	Anjozorobe	Yes	Yes	Yes
449-08	Human	Serum	2008 Feb 26	Anjozorobe	Yes	No	Yes
619-08	Human	Serum	2008 Mar 9	Ankazobe	Yes	Yes	Yes
682-08	Human	Serum	2008 Mar 12	Ambatondrazaka	Yes	Yes	Yes
683-08	Human	Serum	2008 Mar 12	Ambatondrazaka	Yes	Yes	Yes
684-08	Human	Serum	2008 Mar 12	Ambatondrazaka	Yes	Yes	Yes
693-08	Human	Serum	2008 Mar 11	Amparafaravola	Yes	Yes	Yes
776-08	Human	Serum	2008 Mar 14	Ambatondrazaka	Yes	Yes	Yes
779-08	Human	Serum	2008 Mar 16	Amparafaravola	Yes	No	Yes
845-08	Human	Serum	2008 Mar 23	Manjakandriana	Yes	Yes	Yes
846-08	Human	Serum	2008 Mar 23	Manjakandriana	Yes	Yes	Yes
847-08	Human	Serum	2008 Mar 23	Manjakandriana	Yes	Yes	Yes
848-08	Human	Serum	2008 Mar 23	Manjakandriana	Yes	Yes	Yes
849-08	Human	Serum	2008 Mar 23	Manjakandriana	Yes	Yes	Yes
850-08	Human	Serum	2008 Mar 23	Manjakandriana	Yes	Yes	No
851-08	Human	Serum	2008 Mar 23	Manjakandriana	Yes	Yes	Yes
852-08	Human	Serum	2008 Mar 23	Manjakandriana	Yes	Yes	Yes
853-08	Human	Serum	2008 Mar 23	Manjakandriana	Yes	Yes	Yes
854-08	Human	Serum	2008 Mar 23	Manjakandriana	Yes	Yes	Yes
855-08	Bovine	Serum	2008 Mar 24	Manjakandriana	Yes	Yes	Yes
856-08	Bovine	Serum	2008 Mar 24	Manjakandriana	Yes	No	Yes
857-08	Bovine	Serum	2008 Mar 24	Manjakandriana	Yes	No	No
859-08	Bovine	Serum	2008 Mar 24	Manjakandriana	Yes	No	Yes
863-09	Human	Serum	2008 Mar 25	Antananarivo	Yes	Yes	Yes
878-09	Human	Serum	2008 Mar 26	Amparafaravola	Yes	Yes	Yes
879-08	Human	Serum	2008 Mar 26	Manjakandriana	Yes	Yes	Yes
889-08	Human	Serum	2008 Mar 26	Manjakandriana	Yes	Yes	No
892-09	Human	Serum	2008 Mar 26	Manjakandriana	Yes	Yes	Yes
895-09	Human	Serum	2008 Mar 26	Manjakandriana	Yes	Yes	Yes
897-08	Human	Serum	2008 Mar 26	Manjakandriana	Yes	Yes	Yes
1464-08	Human	Serum	2008 Mar 28	Tolagnaro	Yes	Yes	Yes
1585-08	Caprine	Serum	2008 Apr 13	Antsiranana I	Yes	Yes	Yes
1586-08	Human	Serum	2008 Apr 15	Antavy Atsimo	Yes	Yes	Yes
1627-08	Bovine	Serum	2008 Apr 18	Ambalavao	Yes	Yes	Yes
1695-08	Ovine	Serum	2008 Apr 20	Antsiranana II	Yes	Yes	Yes
1730-08	Bovine	Spleen	2008 Apr 25	Fianarantsoa I	Yes	Yes	Yes
2032-08	Human	Serum	2008 May 25	Amparafaravola	Yes	Yes	Yes
2033-08	Human	Serum	2008 Apr 29	Amparafaravola	Yes	Yes	Yes
2034-08	Human	Serum	2008 Apr 23	Amparafaravola	Yes	Yes	Yes
6510-08	Bovine	Liver	2008 Dec 1	Fianarantsoa I	Yes	Not done	Not done
6546-08	Bovine	Liver	2008 Nov 28	Fianarantsoa I	Yes	Not done	Not done
6547-08	Bovine	Liver	2008 Nov 29	Fianarantsoa II	Yes	Not done	Not done
6660-08	Human	Serum	2008 Dec 12	Fianarantsoa I	Yes	Not done	Not done

Partial M sequences (nt positions 781–1536 of the coding domain) and partial L sequences (nt positions 1276–1839 of the coding domain) were available, respectively, from 39 and 42 of the 46 strains from which we obtained the S sequence. Phylogenetic analysis performed as described above, and including the M or L sequences of the strains used for the S analysis, confirmed that these sequences were closer to sequences obtained from Kenya strains circulating in 2006–2007 (data not shown).

**Figure 2:**
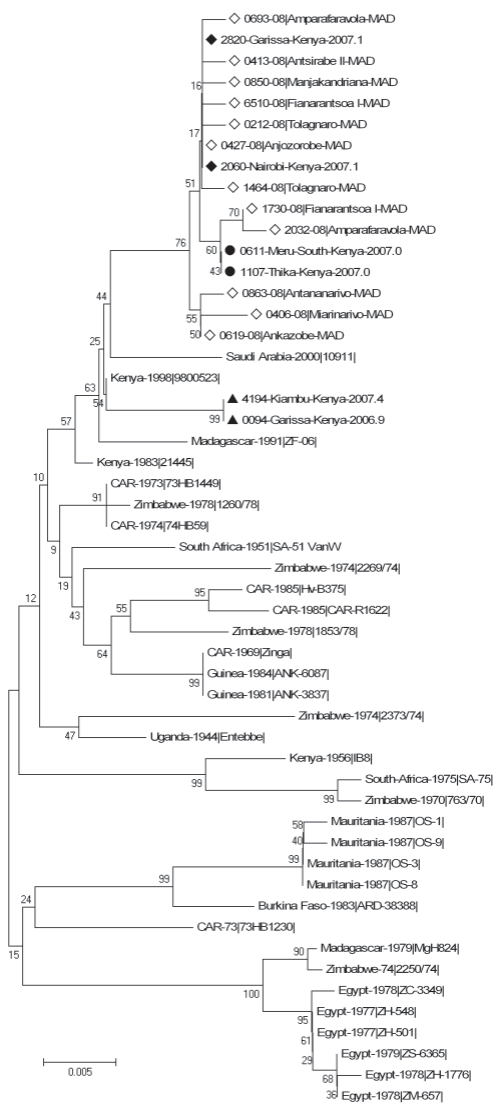
Phylogenetic tree based on the entire small sequences from 33 Rift Valley fever virus strains described by Bird et al. (*16*), from 6 sequences representative of the Kenya 1, 1a, and 2 lineages described by Bird et al. (*18*), and from 1 Madagascar strain circulating in 1991 and 12 Madagascar strains circulating in 2008. Boostrap percentages (from 1,000 resamplings) are indicated at each node. ◆ indicates sequences from the 2006–2007 Kenya-1 lineage, ● indicates sequences from the 2006–2007 Kenya-1a lineage, ▲ indicates sequences from the 2006–2007 Kenya 2 lineage, and ◇ indicates the 2008 Madagascar sequences. Scale bar indicates nucleotide substitutions per site.

Analysis of sequences from strains detected in during the second outbreak November and December 2008 was limited to 4 strains (6510–08, 6546–08, 6547–08, 6660–08) and to the S segment (627 nt). All were close or identical to the Madagascar sequences detected during the first outbreak and clustered within the Kenya-1 and -1a lineages (Figure 2).

### Cross-Sectional Serologic Survey

Confirmed and probable cases were detected in the central highlands and in 2 nonadjoining and well-separated areas, the southern and northern coasts of the island (Figure 1). Geographic distribution of RVFV recent circulation was hypothesized to be larger. To confirm this hypothesis, a nationwide serologic survey was organized. Serum was collected from September 27, 2008, through May 27, 2009, from 1,995 volunteers, who were at high risk for RVFV infection, from 106 of the 111 Malagasy districts. Probable recent RVFV infection was detected for 214 participants, probable past RVFV infection was detected for 219 participants, and recent or past RVFV infection was ruled out for the rest (Appendix Table). Overall (considering the location of the persons with confirmed and probable cases), recent circulation of RVFV was detected in 70 districts and recent or past infection in 92 districts, which indicated wide circulation of RVFV in Madagascar (Figure 1).

## Discussion

Since the first field and laboratory investigations conducted by IPM in the 1970s, 15 arboviruses have been isolated in Madagascar, 10 of which are known to be pathogenic for humans and 3 of which (dengue virus type 1, chikungunya virus, and RVFV) have been responsible for large outbreaks (*4*–*8*,*19*–*22*). The RVF outbreak in 2008–2009 is the largest detected in the country, although during this outbreak the reporting and sampling for suspected cases in humans and animals were not optimal. RVF is a rural disease occurring mainly during the rainy season. Transfer of information from the central administration to the health centers and back was challenging during this period in this low-income country. This lack of recorded information prevented us from describing the outbreak in more detail and estimating the extent of human disease. However, the extent of the disease could be established through a retrospective study in the outbreak area, comparing the crude death rate observed during the outbreak period with an expected death rate computed from data for previous years. The effect on livestock is even more difficult to quantify because of the lack of animal population data and the limited number of specimens submitted for diagnosis.

The addition of the Madagascar RVFV 2008 strains to the Kenya-1 2006–2007 lineage raises the question of this lineage’s introduction from Kenya. We detected IgG against RVF in serum from 18 of 24 goats sampled by the end of June 2008 in Toliara II district (no. 602) on the southeast coast where abortions were reported in early 2007 (J.-M.R., unpub. data). RVFV was probably circulating in this area while it was circulating in Kenya, and perhaps it was introduced from Kenya at that time. Official records document exportation (but no importation) of ruminants from Madagascar to the Comoros archipelago; subsequent exportation to the African continent is possible (L.T.R., pers. comm.). However, illegal importation cannot be excluded. The question of introduction remains unanswered. More complete phylogenetic studies, and full sequences of Madagascar 2008 isolates, are needed to detect the circulation of >1 lineages during the 2008 outbreak and to get a better understanding of the movements and evolution of Madagascar and Kenya isolates.

The nationwide cross-sectional serosurvey supplemented the information obtained during the outbreak. The serologic observations suggest that the virus has probably circulated in the past in most districts and more recently, in 2008–2009, at least in all regions of the country. The sample collection from persons at risk started 3 months and ended 12 months after the detection of the last case of the 2008 outbreak. Despite not having data on duration of IgM against RVFV in humans, we suspect that IgM may have already disappeared in some of the serum samples tested and that the area of recent RVFV circulation is indeed larger than the one we studied. The lack of evidence of virus circulation in some adjoining districts from arid southern Madagascar may be also explained by our small sample size from some of them (Appendix Table). However, the serologic investigation conducted among cattle sampled after the 2008 RVF outbreak indicated that the virus has circulated in the following districts: Midongy-Atsimo (no. 318), Vangaindrano (no. 320), Ampanihy (no. 605), Sakaraha (no. 620), Betioky (no. 612), and Toliara-I. (no. 601) (Elisabeth Jeanmaire, unpub. data) and reduced the area of contiguous districts where the virus circulation was not detected to the following 6 districts: Iakora (no. 311), Befotaka (no. 307), Amboasary-Atsimo (no. 603), Ambovombe-Androy (no. 604), Tsihombe (no. 621), and Bekily (no. 607).

Recent circulation of RVFV in the country was extensive. The detection of the same haplotype from serum sampled at the same period, from the 2 first reported outbreak places 500 km apart may be explained by the large-scale movement of cattle within the country. Observed movements of cattle from rural areas to provincial capitals and between provincial capitals and Antananarivo, could explain the rapid spread of the virus. However, we do not know where the outbreak started; thus, reemergence of RVFV from different places cannot be ruled out. We found the results of some unpublished studies reporting the movement of ruminants in some areas, but a comprehensive study of these movements is needed for a better understanding of the epidemiology of the disease and to organize its surveillance and control.

The geographic distribution of RVF encompasses all 4 ecozones of Madagascar (www.nationalgeographic.com/wildworld/terrestrial.html). This finding suggests that mosquito transmission may occur in all of them. Extensive entomologic studies conducted out in the 1980s in Madagascar have shown that some species described as vectors on the African continent were present in some or all 4 ecozones (*19*). This finding implies that cycles of transmission involving different species may occur in Madagascar. Until now, little information on RVFV vectors in Madagascar has been available (*19*). Thousands of mosquitoes were collected in the highlands during the 2008–2009 outbreaks. The results of the virus detection are still pending and will contribute to the knowledge of the RVFV vectors in Madagascar.

The model used to predict at risk RVF situation has been efficient in the Horn of Africa (*3*,*23*). When this model was applied to Madagascar, the area where probable and confirmed cases were reported was not predicted to be at risk (*24*), suggesting that the model needs to be adjusted for Madagascar. This last point and the questions raised above underline the need for research studies and surveillance on RVF in Madagascar to better predict, declare, and respond to RVF outbreaks.

## Supplementary Material

Appendix Figure Regions and districts of Madagascar, 2008.

Appendix Table Statistical Support for Horizontal Gene Transfer
