# Supplementary figures and images for: Rift Valley Fever during Rainy Seasons, Madagascar, 2008 and 2009
Source: Emerg Infect Dis. 2010 Jun;16(6):963–70. doi: 10.3201/eid1606.091266 (PMC3086256; doi:10.3201/eid1606.091266)

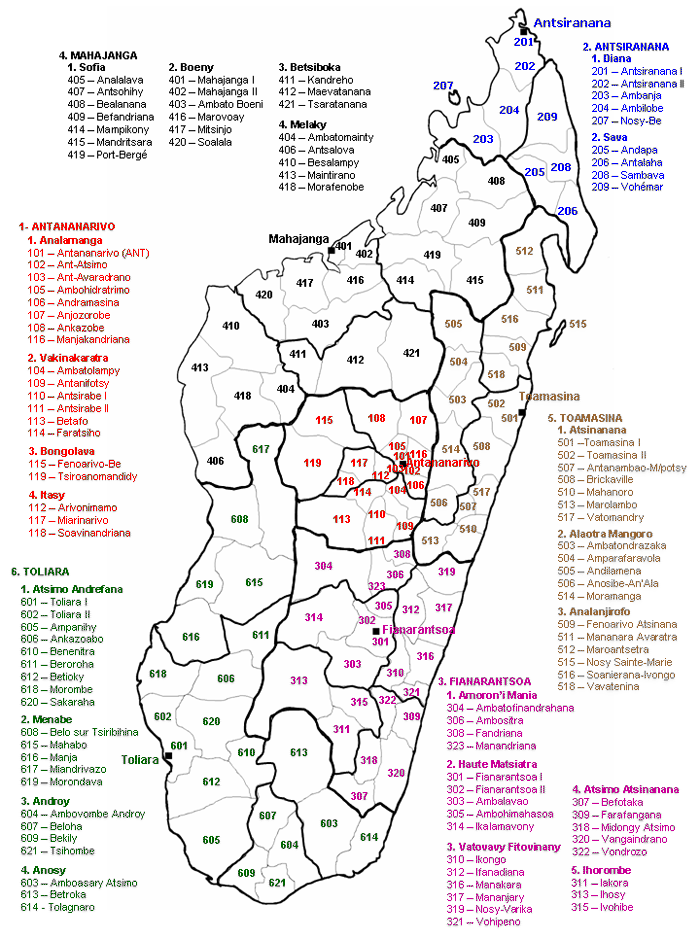

Supplement: Appendix Figure — Regions and districts of Madagascar, 2008. [file 09-1266-appF-s1.gif]
