# Supplementary material for: Rift Valley Fever during Rainy Seasons, Madagascar, 2008 and 2009
Source: Emerg Infect Dis. 2010 Jun;16(6):963–70. doi: 10.3201/eid1606.091266 (PMC3086256; doi:10.3201/eid1606.091266)
Supplement: Appendix Table — Statistical Support for Horizontal Gene Transfer [file 09-1266-appT-s2.pdf]

Appendix Table. Rift Valley fever serologic survey among persons exposed to ruminants in slaughterhouses in the 111 administrative districts of Madagascar, 2008–2009*

Region and district	District code	No infection (IgM– and IgG–)	Past infection (IgM– and IgG+)	Recent infection (IgM+)	Total analyzed	% Risk of sampling only negative samples if prevalence in population is 10%
Analamanga						
Antananarivo	101			No slaughterhouse		
Antananarivo–Atsimo	102	7	0	3	10	35
Antananarivo–Avaradrano	103	13	2	5	20	12
Ambohidratrimo	105	10	1	5	16	19
Andramasina	106	23	1	5	29	5
Anjozorobe	107	10	0	2	12	28
Ankazobe	108	8	2	2	12	28
Manjakandriana	116	3	1	1	5	59
Vakinankaratra						
Ambatolampy	104	23	4	0	27	6
Antanifotsy	109	30	1	1	32	3
Antsirabe I	110	20	0	9	29	5
Antsirabe II	111	11	1	13	25	7
Betafo	113	31	5	14	50	1
Faratsiho	114	17	0	4	21	11
Bongolava						
Fenoarivo-Be	115	18	0	2	20	12
Tsiroanomandidy	119	32	1	1	34	3
Itasy						
Arivonimamo	112	40	9	10	59	0.2
Miarinarivo	117	15	3	0	18	15
Soavinandriana	118	17	1	2	20	12
Diana						
Antsiranana I	201	25	0	5	30	4
Antsiranana II	202	12	0	0	12	28
Ambanja	203	11	1	2	14	23
Ambilobe	204	26	3	4	33	3
Nosy-Be	207	29	1	1	31	4
Sava						
Andapa	205	28	3	3	34	3
Antalaha	206	13	3	1	17	17
Sambava	208	13	13	6	32	3
Vohemar	209	18	4	2	24	8
Amoron'i Mania						
Ambatofinandrahana	304	13	0	0	13	25
Ambositra	306	29	2	0	31	4
Fandriana	308	22	5	3	30	4
Manandriana	323	9	1	0	10	35
Haute-Matsiatra						
Fianarantsoa I	301	33	1	1	35	3
Fianarantsoa II	302	7	2	1	10	35
Ambalavao	303	31	1	1	33	3
Ambohimahasoa	305	14	4	0	18	15
Ikalamavony	314			No sample received		
Vatovavy Fitovinany						
Ikongo	310	7	3	0	10	35
Ifanadiana	312	10	1	1	12	28
Manakara	316	10	3	4	17	17
Mananjary	317	9	4	1	14	23
Nosy-Varika	319	5	1	0	6	53
Vohipeno	321	17	3	4	24	8
Atsimo Atsinanana						
Befotaka	307	7	0	0	7	48
Farafangana	309	12	4	0	16	19
Midongy Atsimo	318	7	0	0	7	48

Vangaindrano	320	6	0	0	6	53
Vondrozo	322	5	1	1	7	48
Ihorombe						
Iakora	311	8	0	0	8	43
Ihosy	313	8	1	0	9	39
Ivohibe	315	1	1	1	3	73
Sofia						
Analalava	405	4	1	1	6	53
Antsohihy	407	3	1	0	4	67
Bealanana	408	17	2	0	19	14
Befandriana	409	8	1	0	9	39
Mampikony	414	23	2	1	26	6
Mandritsara	415	5	1	0	6	53
Port-Bergé	419	17	2	1	20	12
Boeny						
Mahajanga I	401	27	3	3	33	3
Mahajanga II	402	6	0	0	6	53
Ambato Boeni	403	7	0	0	7	48
Marovoay	416	13	0	1	14	23
Mitsinjo	417	5	2	1	8	43
Soalala	420	3	1	0	4	67
Betsiboka						
Kandreho	411	6	2	0	8	43
Maevatanana	412	9	2	2	13	25
Tsaratanana	421	16	1	2	19	14
Melaky						
Ambatomainty	404	21	2	0	23	9
Antsalova	406	9	3	0	12	28
Besalampy	410	13	4	1	18	15
Maintirano	413	23	2	7	32	3
Morafenobe	418	15	4	1	20	12
Atsinanana						
Toamasina I	501	16	10	6	32	3
Toamasina II	502	6	0	4	10	35
Antanambao Manampotsy	507	16	0	1	17	17
Brickaville	508	20	6	4	30	4
Mahanoro	510	6	6	1	13	25
Marolambo	513	No sample received				
Vatomandry	517	16	1	3	20	12
Alaotra Mangoro						
Ambatondrazaka	503	26	1	7	34	3
Amparafaravola	504	13	1	4	18	15
Andilamena	505	21	2	4	27	6
Anosibe-An'ala	506	3	3	2	8	43
Moramanga	514	20	6	7	33	3
Analanjirofo						
Fenoarivo Atsinana	509	31	2	2	35	3
Mananara Avaratra	511	4	0	0	4	67
Maroantsetra	512	25	4	0	29	5
Nosy Sainte Marie	515	16	5	2	23	9
Soaniernana-Ivongo	516	15	3	2	20	12
Vavatenina	518	24	4	2	30	4
Atsimo Andrefana						
Toliara I	601	10	0	0	10	35
Toliara II	602	8	2	5	15	21
Ampanihy	605	3	0	0	3	73
Ankazoabo	606	6	2	0	8	43
Benenitra	610	4	1	0	5	59
Beroroha	611	No samples received				
Betioky	612	No samples received				

Morombe	618	11	1	0	12	28
Sakaraha	620	9	0	0	9	39
Menabe						
Belo Tsiribihina	608	15	4	0	19	14
Mahabo	615	20	6	2	18	15
Manja	616	20	5	2	27	6
Miandrivazo	617	16	1	0	17	17
Morondava	619	22	6	2	30	4
Androy						
Ambovombe Androy	604	31	0	0	31	4
Beloha	607	12	0	0	12	28
Bekily	609	13	1	0	14	23
Tsihombe	621	6	0	0	6	53
Anosy						
Amboasary Atsimo	603	5	0	0	5	59
Betroka	613	21	0	1	22	10
Tolagnaro	614	29	2	9	40	1
Total		1,562	219	214	1,995	

*Ig, immunoglobulin; –, negative; +, positive.
